# Knockdown of PAR2 alleviates cancer-induced bone pain by inhibiting the activation of astrocytes and the ERK pathway

**DOI:** 10.1186/s12891-022-05312-x

**Published:** 2022-05-30

**Authors:** Yiting Tang, Yupeng Chen, Mingzhu Yang, Qiuhui Zheng, Yaoyuan Li, Yanju Bao

**Affiliations:** 1 Department of Oncology, Guang’anmen Hospital, China Academy of Chinese Medical Sciences, Beixiange 5, Xicheng District, 100053 Beijing, China; 2 Department of Hematology and Oncology, Qinghai Provincial Hospital of Traditional Chinese Medicine, No.338 Qiyi Road, Chengzhong District, 810000 Xining, Qinghai Province China

**Keywords:** Cancer-induced bone pain, PAR-2, Astrocytes, ERK, GFAP, PAR-2 inhibitor, p-ERK, p-CREB

## Abstract

**Objective:**

Cancer-induced bone pain (CIBP) is a kind of pain with complex pathophysiology. Proteinase-activated receptor 2 (PAR-2) is involved in CIBP. This study explored the effects of PAR-2 on CIBP rats.

**Methods:**

CIBP rat model was established by injecting Walker 256 rat breast cancer cells into the left tibia of female Sprague-Dawley rats and verified by tibial morphology observation, HE staining, and mechanical hyperalgesia assay. CIBP rats were injected with PAR-2 inhibitor, ERK activator, and CREB inhibitor through the spinal cord sheath on the 13th day after operation. CIBP behaviors were measured by mechanical hyperalgesia assay. On the 14th day after operation, L4-5 spinal cord tissues were obtained. PAR-2 expression, co-expression of PAR-2 and astrocyte marker GFAP, GFAP mRNA and protein levels and the ERK pathway-related protein levels were detected by Western blot, immunofluorescence double staining, RT-qPCR, and Western blot.

**Results:**

CIBP rats had obvious mechanical hyperalgesia and thermal hyperalgesia from the 7th day after modeling; mechanical hyperalgesia threshold and thermal threshold were decreased; PAR-2 was increased in spinal cord tissues and was co-expressed with GFAP. PAR-2 silencing alleviated rat CIBP by inhibiting astrocyte activation. p-ERK/t-ERK and p-CREB/t-CREB levels in CIBP spinal cord were elevated, the ERK/CREB pathway was activated, while the ERK/CREB pathway was inhibited by PAR-2 silencing. The alleviating effect of PAR-2 inhibitor on hyperalgesia behaviors in CIBP rats were weakened by ERK activator, while were partially restored by CREB inhibitor.

**Conclusions:**

PAR-2 knockdown inhibited the ERK/CREB pathway activation and astrocyte activation, thus alleviating CIBP in rats.

**Supplementary information:**

The online version contains supplementary material available at 10.1186/s12891-022-05312-x.

## Introduction

Pain is a kind of multi-faceted sensation existing in the whole nervous system, which often starts in the periphery, where nerve or tissue damage is caused by disease, lesion, or trauma, including tumor growth nerves or tissue [1]. Cancer-induced bone pain (CIBP) is a specific pain condition that includes visceral, inflammatory, and neuropathic pain caused by the metastasis to the bone from the malignant tumors, which is the largest source of the pain of patients with cancer [2]. CIBP is usually a mixture of steady pain and the pain exacerbated by movement or weight-bearing called episodic or incident pain, and it’s commonly seen in patients with primary bone tumors or lung cancer, breast cancer, and prostate cancer [3]. Accumulating evidence has supported that the activation of astrocytes contributes to neuropathic pain development [4]. The treatment for CIBP at present is multi-modal (pharmacological and non-pharmacological), including symptomatic analgesic treatment and causal anti-cancer [5]. However, these therapies are still difficult to control the pain and have obvious adverse reactions. Therefore, exploring more and better methods of early screening and gene diagnosis is of great significance for early diagnosis, timely treatment, and prognosis improvement of CIBP.

Proteinase-activated receptor 2 (PAR-2) is a member of the G-protein coupled receptor family, which is expressed on the cell membrane surface [6]. It is abundantly expressed in the astrocytes [7]. PAR2 protein expression in the spinal cord was upregulated in CIBP rats and it contributes to the hyperalgesia and the transmission and regulation of pain information in a variety of cancers [8]. The mechanism of PAR-2 on CIBP remains to be further explored.

Extracellular signal-regulated kinase (ERK), the main subfamily of mitogen-activated protein kinase (MAPK) family, is critical for extracellular stimuli transduction into the post-translational and the transcriptional responses, which contributes to the pain hypersensitivity related to the nerve and tissue injury [9]. The extracellular signal-regulated kinase (ERK) pathway plays a core role in neuronal plasticity and central sensitization, and ERK phosphorylation activates the transcription factor CREB and CREs as promoters encode pain-related genes [10]. The continuous stage of CREB phosphorylation is mediated by a delayed ERK signal cascade, which is of great significance for the occurrence, development, and maintenance of chronic pain [11]. Activation of the ERK pathway in the spinal cord of CIBP rats is related to astrocyte activation [12]. PAR-2 is reported to regulate inflammatory pain by regulating the ERK pathway [13]. The interaction mechanism of the ERK pathway in CIBP needs to be further studied. However, whether PAR-2 induces CIBP via the ERK/MEK pathway is largely unknown. This study set out to investigate the molecular mechanism of PAR-2 on CIBP, to find a new target for the treatment of CIPB from the perspective of genes.

## Materials and methods

### Ethics statement

 All experimental protocols were approved by the laboratory animal ethics committee of Guang’anmen Hospital (Approval number: IACUC-GAMH-2021-023). All methods were carried out in accordance with relevant guidelines and regulations. The study was carried out in compliance with the ARRIVE guidelines.

## CIBP rat model establishment and grouping

A total of 90 specific pathogen-free (SPF) female Sprague Dawley (SD) rats weighing 150 ± 20 g were purchased from Laboratory Animal Resources, Chinese Academy of Sciences. The rats were raised in the secondary clean animal room in separate cages in a 12-h light/dark cycle at a constant temperature of 23 ± 2℃ with 60-70% humidity and standard feed and freely available water every day. The rats were used for experiments after 3 d of the adaption to the environment. Referring to the previous study, the CIBP rat model was established using the tumor cell implantation (TCI) method [14]. Briefly, the rats were anesthetized with 50 mg/kg pentobarbital sodium by intraperitoneal injection. Rat left leg was shaved and the top half of the tibia was exposed after disinfection with 75% (v/v) ethanol and 7% iodine. Walker 256 cells (3 × 10^5^) were injected slowly into the intramedullary space of the left tibia of the rats, whereas an equivalent volume of Hank’s balanced salt solution (HBSS) was injected into the same position of rats in the sham group using a 50 µl Hamilton microsyringe. The syringe was left in the injection site for another 1 min to prevent tumor cell leakage. The injection site was sealed with bone wax after the syringe was removed. The tibia was radiographically visualized for tumor growth evaluation. In the sham group (*N* = 12), the sham operation was performed, and the cell suspension was replaced with saline of the same volume. After the establishment of the CIBP model, the rats were anesthetized with an intraperitoneal injection of 2% pentobarbital sodium 45 mg/kg, and subarachnoid catheterization was performed. The rats were fed in a single cage after catheterization. The rats with hindlimb lameness, paralysis, local infection, abnormal activity, and catheter detachment were excluded from the experiment.

The model rats were randomly assigned to 9 groups using the random number table method including sham group (*N* = 12), CIBP group (TCI) (*N* = 36), CIBP + PAR-2 inhibitor group (TCI + PI group) (*N* = 6), CIBP + normal saline group (TCI + N group) (*N* = 6), CIBP + PAR-2 control peptide group (TCI + PJ group) (*N* = 6), CIBP + PAR-2 inhibitor + ERK activator group (TCI +PI + EA group) (*N* = 6), CIBP + PAR-2 inhibitor + dimethyl sulfoxide (DMSO) group (TCI + PI + N) (*N* = 6), CIBP + PAR-2 inhibitor + ERK activator + CREB inhibitor group (TCI + PI + EA + CI) (*N* = 6), and CIBP + PAR-2 inhibitor + ERK activator + DMSO group (TCI + PI + EA + N) (*N* = 6). The model rats were intrathecally injected with 10 µL PAR-2 inhibitor FSLLRY-NH2 (1 mmol/L, FSLLRY-NH2, CAS No.245329-02-6, MedChemExpress, Monmouth Junction, NJ, USA) [15], PAR-2 control peptide (LRGISL-NH2, Bachem Inc., Torrance, CA, USA), 10 µL ERK activator Dehydrocorydaline chloride (10 mg/kg, HY-N0674A, MedChemExpress) [16] and 10 µL CREB inhibitor KG-501 (70,485, Sigma-Aldrich, St. Louis, MO, USA) on the 13th d after the operation. The equivalent volume of normal saline (1 mmol/L) and DMSO were set as the corresponding controls. PAR-2 control peptide LRGISL-NH2 was a reverse PAR2 inactivation peptide [17] that cannot activate PAR2, which was used as the control of PAR-2 inhibitory peptide FSLLRY-NH2.

The hyperalgesia behavior of rats in the sham group and TCI group was evaluated by pain examination on the 3rd, 5th, 7th, 14th, and 21st d after the operation. After hyperalgesia behavior evaluation, the rats were euthanized by intraperitoneal injection of 3% pentobarbital sodium (150 mg/kg), and the L4-5 spinal cord tissues of 30 rats were collected for tissue homogenate (the tissues were collected on the 3rd, 5th, 7th, 14th and 21st day after the operation, with 6 collected at each time point) and then Western blot (WB) and reverse transcription quantitative polymerase chain reaction (RT-qPCR) were performed, and the tibial bone marrow tissues of 6 rats were collected for paraffin embedding after fixing with 4% paraformaldehyde (the tissues were collected on the 14th day after the operation). The hyperalgesia behavior of rats in other groups was evaluated on the 3rd, 5th, 7th, 14th, and 21st d after the operation and the L4-5 spinal cord tissues of rats were collected on the 14th d and made into homogenate, and then, WB and RT-qPCR were performed.

### Mechanical pain threshold determination in rats

The rats were acclimated in plastic cages for 30 min and measured when they were quiet. The test probe (blunt needle) was aligned to the middle part of the hindlimb pelma of the rats. The pelma was pressed using the probe at a uniform increasing speed. When the foot was rapidly retracted, the machine showed the pressure applied to the pelma at this time. The test was repeated 3 times at an interval of 5 min. The mean value was calculated as the mechanical pain threshold with the maximum pressure being 50 g.

### Thermal hyperalgesia determination in rats

The plexiglass box was placed on a glass plate with a thickness of 3 mm, and the rats were placed in the box for adaptation for 30 min. The pelma of rats was irradiated with a thermal stimulator according to the Hargreaves method. The paw withdrawal latency (PWL) was the period from the beginning of irradiation to the occurrence of leg raising avoidance. The automatic cut-off time was set to 20 s to prevent tissue damage. The determination for each rat was repeated 5 times with an interval of at least 3 min. The PWL value was obtained as the mean value of the last 3 times.

## Hematoxylin and eosin (HE) staining

The bone marrow of rat tibia was fixed with 4% polyformaldehyde, dehydrated with conventional gradient ethanol, cleared with xylene, soaked in wax, embedded with paraffin, cut into 4 μm-thick sheets, placed on the glass slides, baked at 60℃ for 2 h and stored at a dry and dark place. The sections were dewaxed with xylene and dehydrated with gradient ethanol. The samples were stained with HE staining kits (G1120, Solarbio, Beijing, China), dehydrated with gradient, cleared with xylene, and sealed with neutral balsam. The morphological and quantitative changes of cells in tibial bone marrow were observed under a Nikon Tioptical microscope (Nikon, Tokyo, Japan).

## Immunofluorescence double labeling

The L4-5 spinal cord tissues of rats were made into paraffined sections. The sections were placed into 0.25% Triton X-l00 solution in an incubator at 37 °C. After rinsing with 0.01 mol/L PBS buffer, the sections were added with 5% donkey serum, sealed for 1 h, and incubated with the mixture of anti-PAR2 (ab180953, 1:1000, Abcam, Cambridge, MA, USA) and astrocyte marker anti-GFAP (ab190288, 1:1000, Abcam) in a wet box at 4℃ overnight. After washing with PBS, the samples were added with fluorescent secondary antibodies goat anti-rabbit IgG H&L (ab6721, 1:2000, Abcam) and goat anti-rat IgG H&L (ab133470, 1:2000, Abcam), incubated at 37 °C for 30 min, washed with PBS and sealed with 4’,6-diamidino-2-phenylindole (DAPI) dye containing anti-fluorescence quenching agent. The automatic bright field/fluorescence microscope and Q550A image analysis system were used to collect images and IPP6.0 image analysis software was used for analysis.

### RT-qPCR

The L4-5 spinal cord tissues of rats were collected. The total RNA of the samples was extracted using TRIzol (Invitrogen, Carlsbad, CA, USA). The 5 µL total RNA sample was diluted with RNase ultrapure water to 20-fold and the optical density (OD) values at 260 and 280 nm of the UV spectrophotometer were read to determine the RNA concentration and purity. The ratio of OD260/OD280 between 1.7 and 2.1 indicated a high purity, which could meet the needs of subsequent experiments. The cDNA template was synthesized by reverse transcription reaction on a PCR amplification instrument. Real-time quantitative RT-PCR was performed using an ABI7500 quantitative PCR instrument. The reaction conditions were pre-denaturation at 95 °C for 10 min and 40 cycles of denaturation at 95 °C for 10 s, annealing at 60 °C for 20 s and extending at 72 °C for 34 s. The threshold cycle (Ct) value of each reaction tube was obtained by manually selecting the threshold value at the lowest point of parallel rise of each logarithmic amplification curve. The data were analyzed using the 2^−ΔΔCt^ method, which presented the ratio of target gene expression between the experimental group and the control group. The formula was as follows: ΔΔCt = [Ct _(target gene)_ - Ct _(internal reference gene)_] _experimental group_ - [Ct _(target gene)_ – Ct _(internal reference gene)_] _control group._ Ct was the number of amplification cycles when the real-time fluorescence intensity of the reaction reached the set threshold. At this time, the amplification increased logarithmically. The experiment was conducted 3 times. The amplification primer sequences of each gene and its primers are shown in Table 1.

**Table 1:** Primer sequences of PAR-2 and GFAP

Name of primer	Sequences
*PAR-2* -F	GCUCCCUGAGACCCUAAC
*PAR-2*-R	CAGTGCAGGGTCCGAGGT
GFAP-F	CTGAGAACGAGGCTGGGTAG
GFAP-R	CCTGAAGGGTGTCAACAGGT
GAPDH-F	GTCGATGGCTAGTCGTAGCATCGAT
GAPDH-R	TGCTAGCTGGCATGCCCGATCGATC

### WB

The L4-5 spinal cord tissues of rats were ground in liquid nitrogen. After washing with pre-cooled PBS buffer 3 times, the samples were lysed with protein extraction lysate of 100 µL/50 mL culture bottle and centrifuged for 10 min. The supernatant was harvested into a 0.5 mL bottle, centrifuged, and stored at -20 °C. The protein concentration was quantified using the bicinchoninic acid (BCA) kit (Pierce, Rockford, IL, USA) and the concentration was adjusted to 2 µg/µL. The diluted standard and samples were added to the test well at 10 µL/well, incubated at 37 °C for 30 min, and cooled to room temperature. The OD value of the samples was determined at the wavelength of 495 nm using an enzyme standard instrument. The standard curve was drawn and the protein concentration was calculated. The samples were stored at -70 °C. The sodium dodecyl sulfate polyacrylamide gel was prepared for protein electrophoresis. After electrophoresis, the protein was transferred to polyvinylidene fluoride membranes using the wet electric transfer method for 2 h. Then, the membranes were sealed with 5% skim milk-tris buffered saline-Tween20 (TBST) and incubated for 1-2 h. After sealing, the membranes were placed in an incubator, added with primary antibodies anti-PAR2 (ab180953, 1:1000, Abcam), anti-GFAP (ab190288, 1:1000, Abcam), anti-ERK (ab184699, 1:10000, Abcam), anti-p-ERK (sc-81,492, 1:2000, Santacruze Biotechnology, Inc., Texas, USA), anti-CREB (9197, 1:1000, Cell signaling technology, USA) and anti-p-CREB (9198, 1:1000, Cell signaling technology, USA) at 4 °C overnight. The samples were washed with TBST 3 times for 10 min each time, added with horseradish peroxidase-labeled secondary antibody goat anti-rabbit IgG (ab6721, 1:2000, Abcam) for 1 h, and washed with TBST 3 times with 10 min for each time. Chemiluminescence, X-ray film compression, development, fixing, and data analysis were performed.

### Co-immunoprecipitation

The L4-5 spinal cord tissues of rats were made into homogenate, centrifuged and the total protein was extracted. The protein concentration was determined using the BCA method and adjusted to 1 g/L. The samples were centrifuged and the supernatant was collected. The moderate amount of Protein A-Agarose was washed with PBS and made into 50% suspension. The samples were added with the known protein antibody anti-PAR2 (ab180953, 1:1000, Abcam) (5 µg) and the precipitation of specific antigen-antibody immune complex and the supernatant was collected. Protein A-Agarose antibody-protein complex was washed with pre-cooled PBS and centrifuged and the supernatant was collected. SDS-PAGE was prepared for protein electrophoresis. The standard and sample were transferred to the PVDF membranes using the wet electric transfer method. The membranes were sealed with 5% skim milk prepared by TBST, incubated with anti-ERK (ab184699, 1:10000, Abcam) on a shaker for 2 h, and incubated with secondary antibody (ab6721, 1:2000, Abcam) for 1 h. Development and fixing were performed and Quantity One software was used for data analysis.

## Statistical analysis

GraphPad Prism 8.01 (GraphPad Software Inc San Diego, CA, USA) and SPSS 21.0 (IBM Corp. Armonk, NY, USA) were used for data analysis and mapping. The data were expressed as mean ± standard deviation. An independent *t* test was used for comparisons between 2 groups. One-way analysis of variance (ANOVA) was used for comparisons among multi-groups. Tukey’s multiple comparisons test was used for the post hoc test. *P* value was obtained by a bilateral test. *P* < 0.05 was indicative of statistical significance.

## Results

### CIBP rat model establishment

**Fig. 1:**
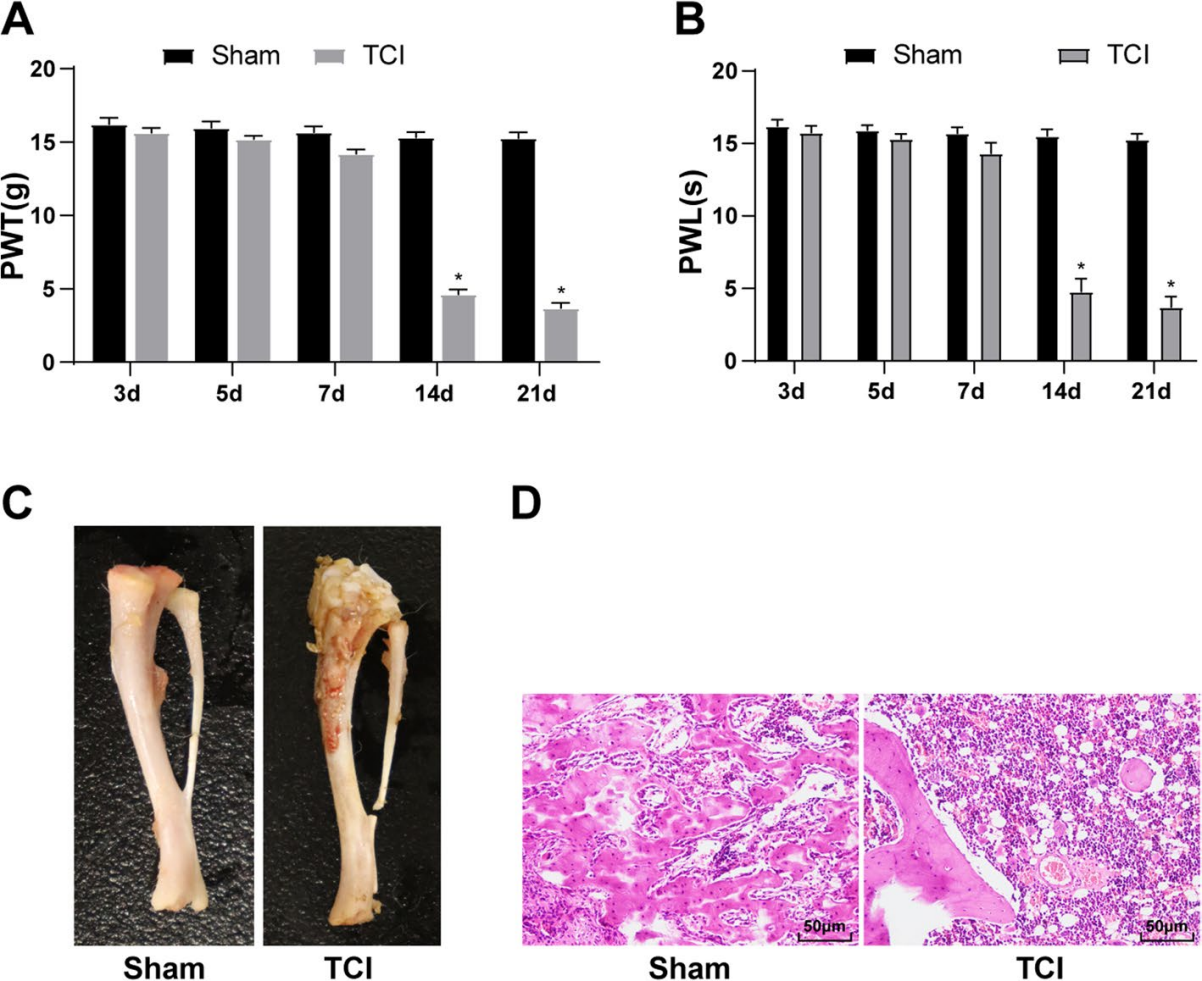
CIBP model establishment in rats. The CIBP rat model was established using Walker 256 rat breast cancer cells. On the 3rd, 5th, 7th, 14^th,^ and 21st d after TCI, **A** The changes of mechanical pain threshold were detected; **B** The changes of thermal hyperalgesia threshold were detected. The tibia of rats was collected 14 d after the TCI. **C** The morphological changes of the tibia were observed; **D** The changes of the tumor cell number in the tibial medullary cavity of rats were observed by HE staining. *N* = 6. The data were expressed as mean ± standard deviation. Independent *t* test was used for comparisons of data in panels A-B. *P < 0.05

First, Walker 256 rat breast cancer cells were injected into the tibial bone marrow cavity of SD rats for TCI to establish a rat model of CIBP. Hyperalgesia behavior test demonstrated that compared with the sham group, there were no evident changes in the mechanical pain threshold and thermal hyperalgesia threshold of TCI-implanted rats during the 0-7 days, while the mechanical pain threshold and thermal hyperalgesia threshold were significantly decreased on the 14th and 21st days (Fig. 1 A-B), and the rats showed obvious mechanical hyperalgesia and thermal hyperalgesia. The morphology of the tibia was observed 14 d after TCI. Compared with the sham group, the rat tibia was obviously destroyed in the TCI group (Fig. 1 C). HE staining showed that in the TCI group, the bone marrow cavity of the tibia was filled with cancer cells 14 d after the TCI (Fig. 1D). These results suggested that the CIBP rat model was successfully established.

## PAR-2 was involved in the formation of hyperalgesia in rat CIBP

**Fig. 2:**
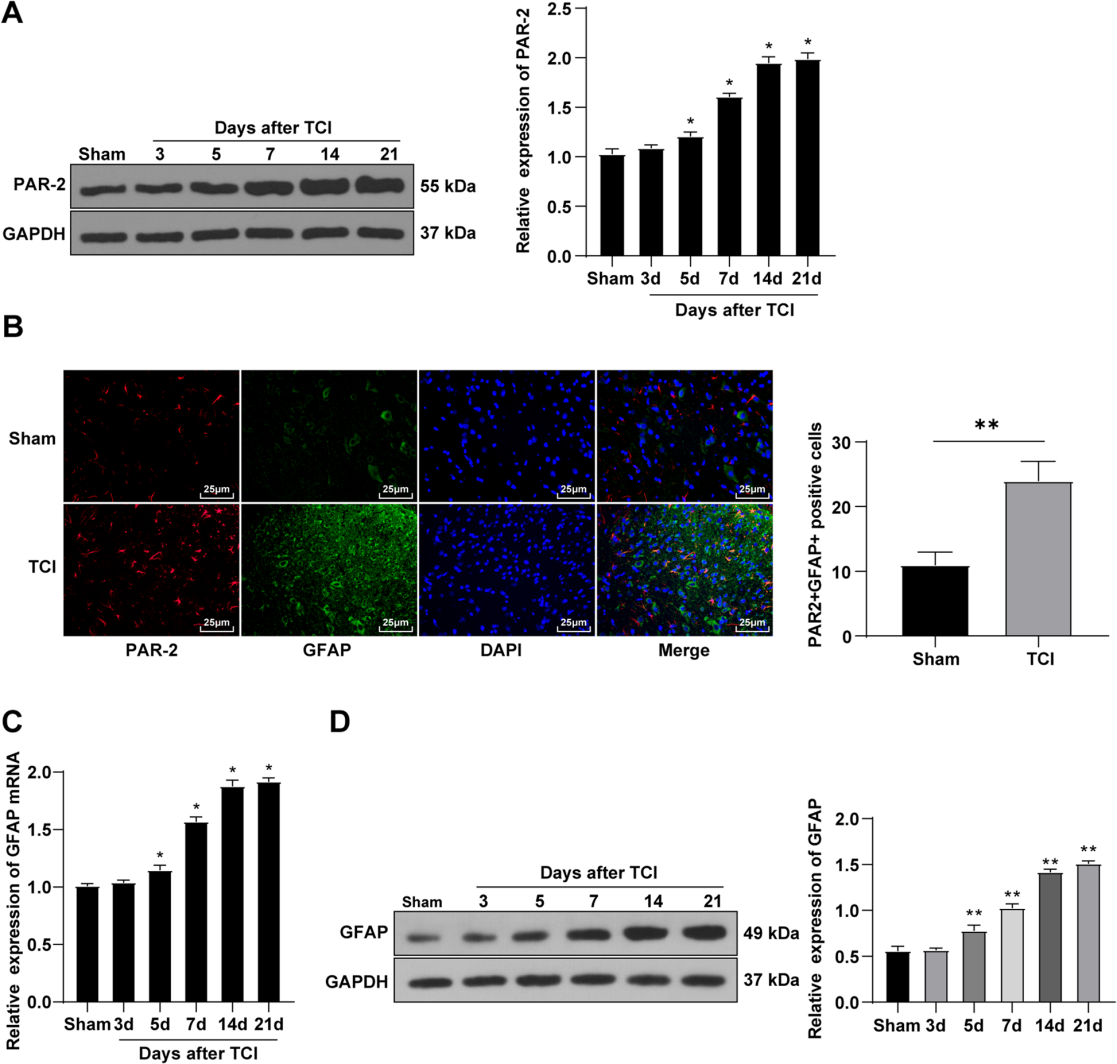
The expression of PAR-2 in the spinal cord of TCI rats was upregulated. On the 3rd, 5th, 7th, 14th and 21st day after modeling, the L4-5 spinal cord tissues were obtained. **A** The expression of PAR-2 in the spinal cord of rats was detected by WB; **B** On the 14th day, the co-expression of PAR-2 and spinal astrocytes was detected by immunofluorescence double staining (the scale was 25 μm); **C** The mRNA level of astrocyte marker GFAP in rat spinal cord was observed by RT-qPCR; **D** The astrocyte marker GFAP protein level in rat spinal cord was detected by WB. *N* = 6. The data were expressed as mean ± standard deviation. One-way ANOVA was used for comparisons of data in panel A-D. Tukey’s multiple comparisons test was used for post hoc test *Compared with the sham group, *P* < 0.05, **Compared with the sham group, *P* < 0.01

The activation of the astrocytes can induce chronic pain and the inhibition of astrocyte function can block the occurrence of chronic pain or relieve the pain [18]. PAR-2 is a subtype of protease-activated receptors (PARs), which is widely expressed in the central nervous system and is highly-expressed in activated astrocytes [8]. PAR-2 is involved in hyperalgesia and the transmission and regulation of pain information in a variety of cancers [19]. To study the effect of PAR-2 on CIBP in rats, the L4-5 spinal cord of rats was collected on the 3rd, 5th, 7th, 14th, and 21st d after modeling. The PAR-2 expression was detected by WB. Compared with the sham group, after TCI induction, the PAR-2 in the spinal cord of rats was upregulated and positively correlated with time and the change started from the 5th day after the operation with a gradual increase on 7-14 days after the operation and lasted until the 21st day (Fig. 2 A). The localization of PAR-2 in spinal astrocytes was detected using the immunofluorescence double staining technique on the 14th day after modeling. PAR-2 was co-expressed with spinal cord astrocyte marker GFAP (Fig. 2B). Furthermore, the mRNA and protein levels of astrocyte marker GFAP were detected by RT-qPCR and WB. Compared with the sham group, the GFAP mRNA and protein levels in the spinal cord of the TCI group were upregulated from the 5th day and were still upregulated until the 21st day (Fig. 2 C-D). Based on these results, we speculated that PAR-2 might play a role in the hyperalgesia of CIBP in rats by inducing the activation of spinal astrocytes.

### Knockdown of PAR-2 inhibited the activation of astrocytes and alleviated CIBP in rats

**Fig. 3:**
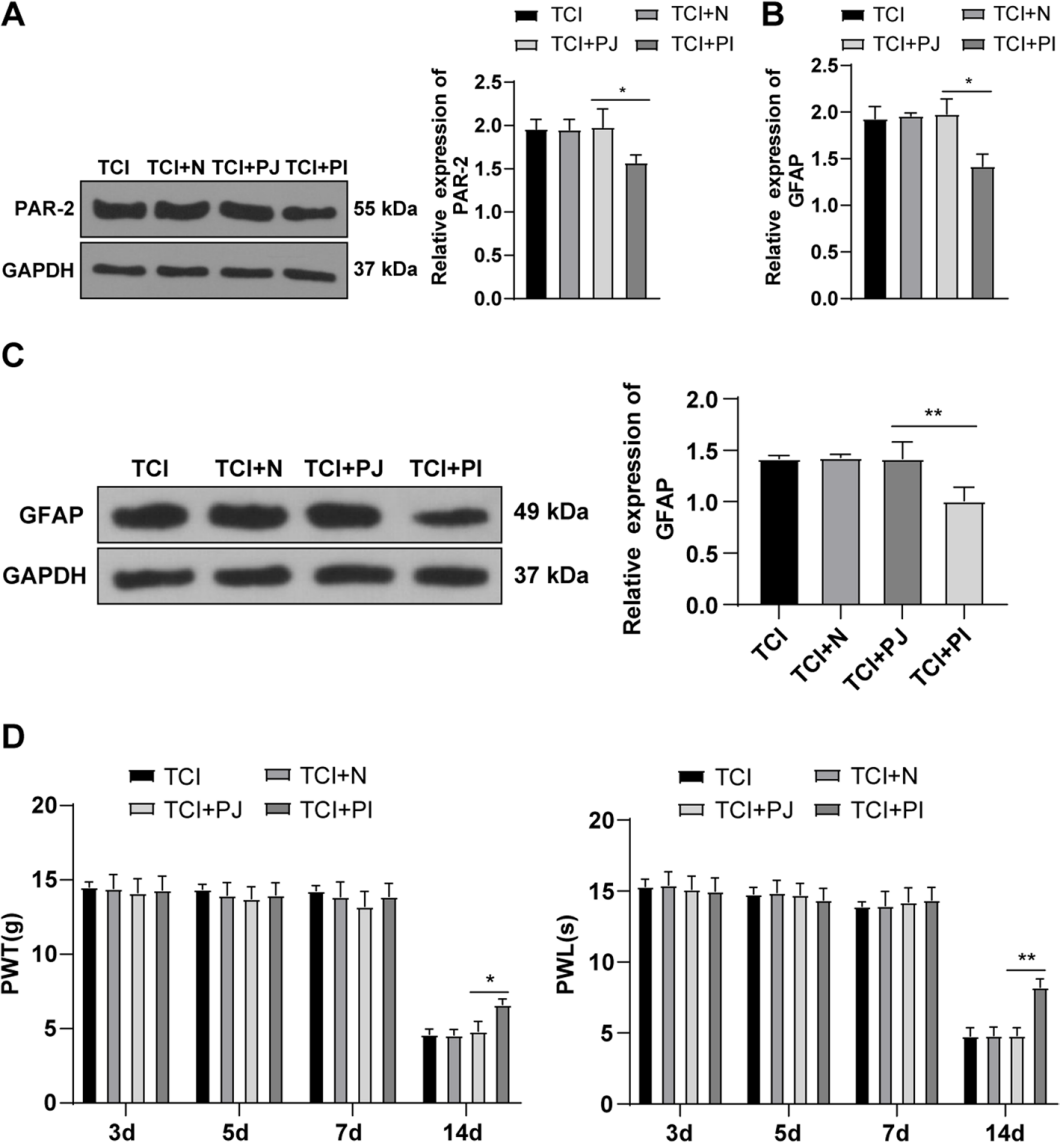
Knockdown of PAR-2 inhibited the activation of GFAP to alleviate CIPB in rats. The L4-5 spinal cord was obtained on the 14th day after modeling. **A** The expression of PAR-2 was detected by WB; **B** The mRNA level of astrocyte marker GFAP was detected by RT-qPCR; **C** The protein level of GFAP was detected by WB; **D** The changes of mechanical pain threshold and thermal pain threshold were detected on the 3rd, 5th, 7th and 14th day. *N* = 6. The data were expressed as mean ± standard deviation. One-way ANOVA was used for comparisons of data among multi-groups. Tukey’s multiple comparisons test was used for post hoc test *P < 0.05, **P < 0.01

To further explore the role of PAR-2 in rat CIBP, administration of PAR-2 control peptide (TCI + PJ) or PAR-2 inhibitor (TCI + PI) was given to the spinal cord of TCI-injected rats. The PAR-2 expression in the L4-5 spinal cord of rats on the 14th day was detected by WB. Compared with the TCI + N group, the PAR-2 in spinal cord tissues of rats in the TCI + PI group was significantly downregulated and the expression wasn’t changed in the TCI + PJ group (Fig. 3 A), indicating that PAR-2 inhibitor successfully downregulated the expression of PAR-2. To further study the action of PAR-2 knockdown on astrocytes, the expression of astrocyte marker GFAP was detected by RT-qPCR and WB. Compared with the TCI + N group, the GFAP in spinal cord tissues of rats in the TCI + PI group was significantly downregulated (Fig. 3B-C), indicating that knockdown of PAR-2 inhibited the activation of astrocytes. In addition, rat hyperalgesia detection demonstrated that repeated administration of PAR-2 control peptide did not improve the hyperalgesia behavior of CIBP rats, while administration of PAR-2 inhibitor PI in the spinal cord could delay or alleviate the CIBP rat hyperalgesia behavior (Fig. 3D). These results suggested that knockdown of PAR-2 inhibited activation of astrocytes, thus alleviating CIBP in rats.

### Knockdown of PAR-2 inhibited the activation of the ERK/CREB pathway

**Fig. 4:**
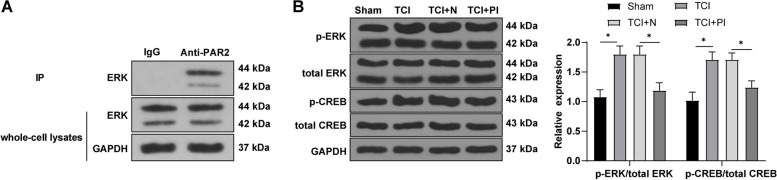
Knockdown of PAR-2 inhibited the activation of the ERK pathway in the spinal cord of the TCI rats. The L4-5 spinal cord tissues were obtained from the CIBP rats on the 14th day. **A** The co-immunoprecipitation of PAR-2 and ERK in spinal astrocytes was detected by immunoprecipitation assay; **B** The levels of the ERK pathway-related proteins were detected by WB. *N* = 6. The data were expressed as mean ± standard deviation. Independent *t* test was used for comparisons of data between 2 groups. *P < 0.05. t-CREB: total CREB; t-ERK: total ERK

The previous study revealed that the ERK pathway activation in the spinal cord of rats with CIBP may be related to astrocyte activation [12]. p-ERK can directly or indirectly phosphorylate some key structures, such as the phosphorylation of transcription factors [20]. The upregulation of p-cAMP response element-binding protein (p-CREB) expression can promote hyperalgesia [21]. Since both PAR-2 and ERK receptors were located on the cell surface to send signals, we assumed that PAR2 could physically interact with ERK receptors to enhance their activity. The L4-5 spinal cord tissues of rats on the 14th day were collected. The relationship between PAR-2 and ERK in rat spinal cord was detected by immunoprecipitation. ERK could be precipitated by PAR-2 antibody in rat spinal cord (Fig. 4 A), indicating that there was an interaction between PAR-2 and ERK in rat spinal cord tissues. Furthermore, the levels of ERK pathway-related proteins in the spinal cord tissues of CIBP rats were detected on the 14th day. Compared with the sham group, the levels of p-ERK/t-ERK and p-CREB/t-CREB in the spinal cord of rats in the TCI group were apparently upregulated, while these levels were significantly downregulated after inhibition of PAR-2 in the TCI rats (Fig. 4B). The results above suggested that knockdown of PAR-2 inhibited the activation of the ERK/CREB pathway in the spinal cord of the TCI rats.

### Knockdown of PAR-2 inhibited the activation of astrocytes and alleviated CIBP in rats by inhibiting the ERK/CREB pathway

**Fig. 5:**
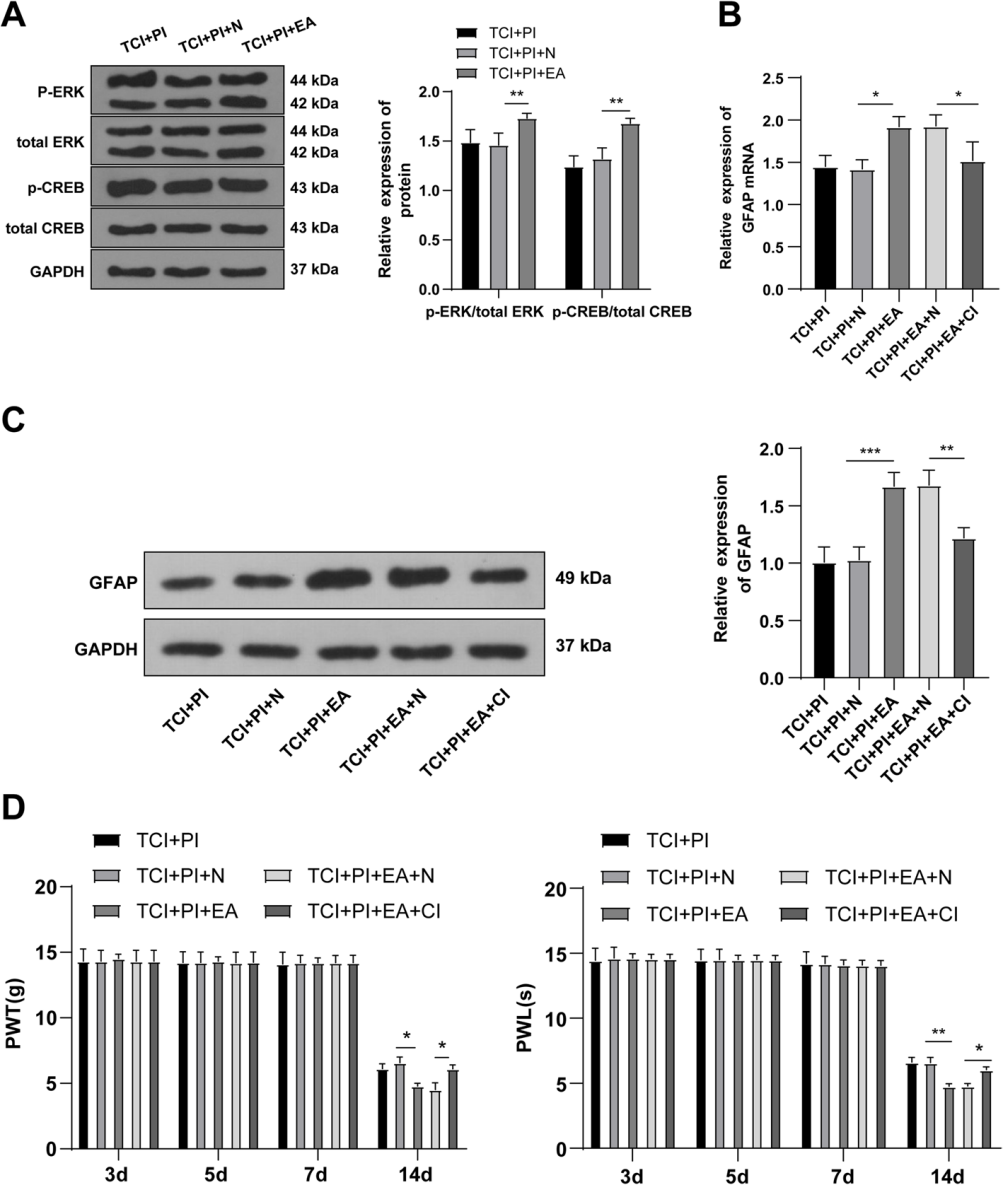
Knockdown of PAR-2 alleviated CIBP by inhibiting the activation of the ERK pathway and inhibiting the activation of astrocytes. The L4-5 spinal cord tissues of TCI rats on the 14th day after injection of PAR-2 inhibitor and ERK activator were obtained. **A** The levels of the ERK pathway-related proteins were detected by WB; **B** The mRNA level of GFAP was detected by RT-qPCR; **C** The protein level of GFAP was detected by WB; **D** The changes of mechanical pain threshold and thermal pain threshold were detected on the 3rd, 5th, 7th and 14th day. *N* = 6. The data were expressed as mean ± standard deviation. Independent *t* test was used for comparisons of data between 2 groups. *P < 0.05, **P < 0.01, ***P < 0.001. t-CREB: total CREB; t-ERK: total ERK

To further explore whether PAR-2 played a role in CIBP by affecting the ERK pathway, the TCI rats administrated with PAR-2 inhibitor were injected with ERK activator Dehydrocorydaline chloride. The levels of the ERK pathway-related proteins in the L4-5 spinal cord tissues of rats on the 14th day were detected. Compared with the TCI + PI + N group, the levels of p-ERK/t-ERK and p-CREB/t-CREB in spinal cord tissues of rats in the TCI + PI + EA group were significantly upregulated (all *P* < 0.05, Fig. 5 A). The expression of the astrocyte marker GFAP was detected by RT-qPCR and WB. Compared with the TCI + PI + N group, the expression of the astrocyte marker GFAP in spinal cord tissues of rats in the TCI + PI + EA group were significantly upregulated (all *P* < 0.05, Fig. 5B-C), indicating that the effect of PAR-2 knockdown on inhibiting astrocyte activation was reversed by ERK activator Dehydrocorydaline chloride. Hyperalgesia behaviors demonstrated that compared with the alleviated or delayed hyperalgesia of rats in the TCI + PI + N group, the mechanical hyperalgesia of the rats in the TCI + PI + EA group could weaken these effects (Fig. 5D). To explore the relationship between PAR-2 and CREB, TCI rats treated with PAR-2 inhibitor and ERK activator were injected with CREB inhibitor. Similarly, the L4-5 spinal cord tissues of rats on the 14th d were collected to detect the expression of GFAP by RT-qPCR and WB. Compared with the TCI + PI + EA + N group, the addition of CREB inhibitor (TCI + PI + EA + CI group) decreased the level of GFAP (all *P* < 0.05, Fig. 5B-C) and promoted hyperalgesia of CIBP rats (*P* < 0.05, Figure 5 D). These results suggested that knockdown of PAR-2 inhibited the activation of astrocytes by inhibiting the ERK/CREB pathway, thus alleviating CIBP in rats.

## Discussion

CIBP is a most difficult pain condition [22]. It is estimated that approximately 60–84% of cancer patients are suffering from different degrees of bone pain [5]. A previous study has revealed that PAR-2 is involved in the alleviation of CIBP [23]. Our study elucidated that the knockdown of PAR-2 inhibited the activation of the ERK pathway and the activation of the astrocytes, thus alleviating CIBP in rats.

Compared with males, females are more sensitive to pain and have lower tolerance. Therefore, in this study, we selected female SD rats as the research subject. PAR-2 is involved in the pain induced by a variety of diseases including irritable bowel syndrome and cancers [24, 25]. Our results demonstrated that PAR-2 expression in the spinal cord of rats after TCI induction was upregulated and was positively correlated with time. Astrocyte activation causes chronic pain and the inhibition of astrocyte activation blocks the occurrence of chronic pain or relieves the pain [18]. Our results showed that PAR-2 was co-expressed with spinal cord astrocyte marker GFAP; GFAP mRNA level in the spinal cord after TCI induction was upregulated from the 5th day and was still upregulated until the 21st day. However, after adding PAR-2 inhibitor in TCI-induced rats, we observed that the hyperalgesia of rats was enhanced, which meant that inhibiting PAR-2 activity could improve hyperalgesia. It is consistent that PAR2 was increased significantly in the dorsal root ganglia neurons, which indicated that the increased PAR2 expression contributes to the thermal hyperalgesia and mechanical allodynia development related to CIBP and PAR2 may be a novel target of pain treatment in CIBP patients [26]. In conclusion, PAR-2 might play a role in the hyperalgesia of CIBP in rats by inducing the activation of spinal astrocytes.

To further study the effects of PAR-2 on CIBP, we repeatedly gave PAR-2 inhibitor administration to the spinal cord of rats after TCI injection. Our results elicited that PAR-2 expression in spinal cord tissues of rats after TCI induction and PAR-2 inhibitor treatment was downregulated and the expression wasn’t changed in the rats after PAR-2 control peptide treatment. GFAP is the major intermediate filament protein of the astrocytes [27]. GFAP expression in spinal cord tissues of rats after PAR-2 inhibitor treatment was downregulated, which indicated that PAR-2 knockdown inhibited astrocyte activation. Besides, repeated administration of PAR-2 control peptide did not improve the hyperalgesia behavior of CIBP rats, while repeated administration of PAR-2 inhibitor delayed or alleviated CIBP. Consistently, the inhibition of PAR-2 suppresses the activation of astrocytes in acute ischemic cerebral [28]. PAR-2 inhibitor alleviates the cancer pain in oral cancer [25]. In brief, knockdown of PAR-2 inhibited activation of astrocytes, thus alleviating CIBP in rats.

The astrocytes can be activated via the ERK pathway [29]. Our results showed that ERK could be precipitated by PAR-2 antibody in rat spinal cord, which indicated that there was an interaction between PAR-2 and ERK in rat spinal cord tissues. CREB phosphorylation in the spinal cord contributes to pain hypersensitivity [30]. Our results demonstrated that p-ERK/t-ERK and p-CREB/t-CREB levels in the spinal cord of TCI rats were significantly upregulated, while the levels were downregulated after PAR-2 inhibition. Consistently, p-ERK and p-CREB activations were increased in CIBP rats [31]. However, there is no report on the effect of PAR-2 on the phosphorylation of ERK. Our study initially suggested that PAR-2 knockdown inhibited the ERK pathway activation in the spinal cord of the TCI rats.

To further study whether PAR-2 had an effect on CIBP by affecting the ERK pathway, we treated TCI rats with PAR-2 inhibitor and injection of ERK activator dehydrocorydaline chloride. Our results showed that p-ERK/t-ERK and p-CREB/t-CREB levels and GFAP expression in spinal cord tissues of rats after ERK activator treatment were upregulated, which indicated that the effect of the knockdown of PAR-2 on inhibiting astrocyte activation was reversed by the ERK activator. The activation of the ERK pathway promotes astrocyte activation and increases GFAP level [32]. Compared with the alleviated or delayed hyperalgesia of rats after PAR-2 inhibitor treatment, the mechanical hyperalgesia of the rats after ERK activator treatment could weaken these effects. Meanwhile, we treated CIBP rats with PAR-2 inhibitor, ERK activator, and CREB inhibitor, and found that the addition of CREB inhibitor repressed the GFAP activity increased by ERK activator, thus alleviating the CIBP symptoms of rats. Collectively, PAR-2 knockdown inhibited astrocyte activation by inhibiting the ERK pathway, thus alleviating CIBP in rats.

In summary, this study supported that the knockdown of PAR-2 inhibited the activation of the ERK pathway and the activation of astrocytes, thus alleviating CIBP in rats. However, there is no in-depth study on the effect mechanism of PAR-2 on the ERK signal transduction in other tissues and cells. Whether different gender has an impact on the mechanism of PAR2-mediated MEK/ERK pathway in CIBP is a limitation of our study, and we will further carry out relevant research in the future. Whether PAR-2 can be used as a target gene for the treatment of CIBP still needs further evidence. Further work is needed to further study the mechanism of PAR-2 on the ERK signal transduction in other tissues and cells, to find a new target for the treatment of CIBP from the perspective of genes.

## Supplementary information

**Additional file 1.**

**Additional file 2.**

**Additional file 3.**

**Additional file 4.**

**Additional file 5.**

**Additional file 6.**

**Additional file 7.**

**Additional file 8.**

## Data Availability

The datasets supporting the conclusions of this article are included within the article (and its additional files).
