# Supplementary figures and images for: Knockdown of PAR2 alleviates cancer-induced bone pain by inhibiting the activation of astrocytes and the ERK pathway
Source: BMC Musculoskelet Disord. 2022 May 30;23:514. doi: 10.1186/s12891-022-05312-x (PMC9150294; doi:10.1186/s12891-022-05312-x)

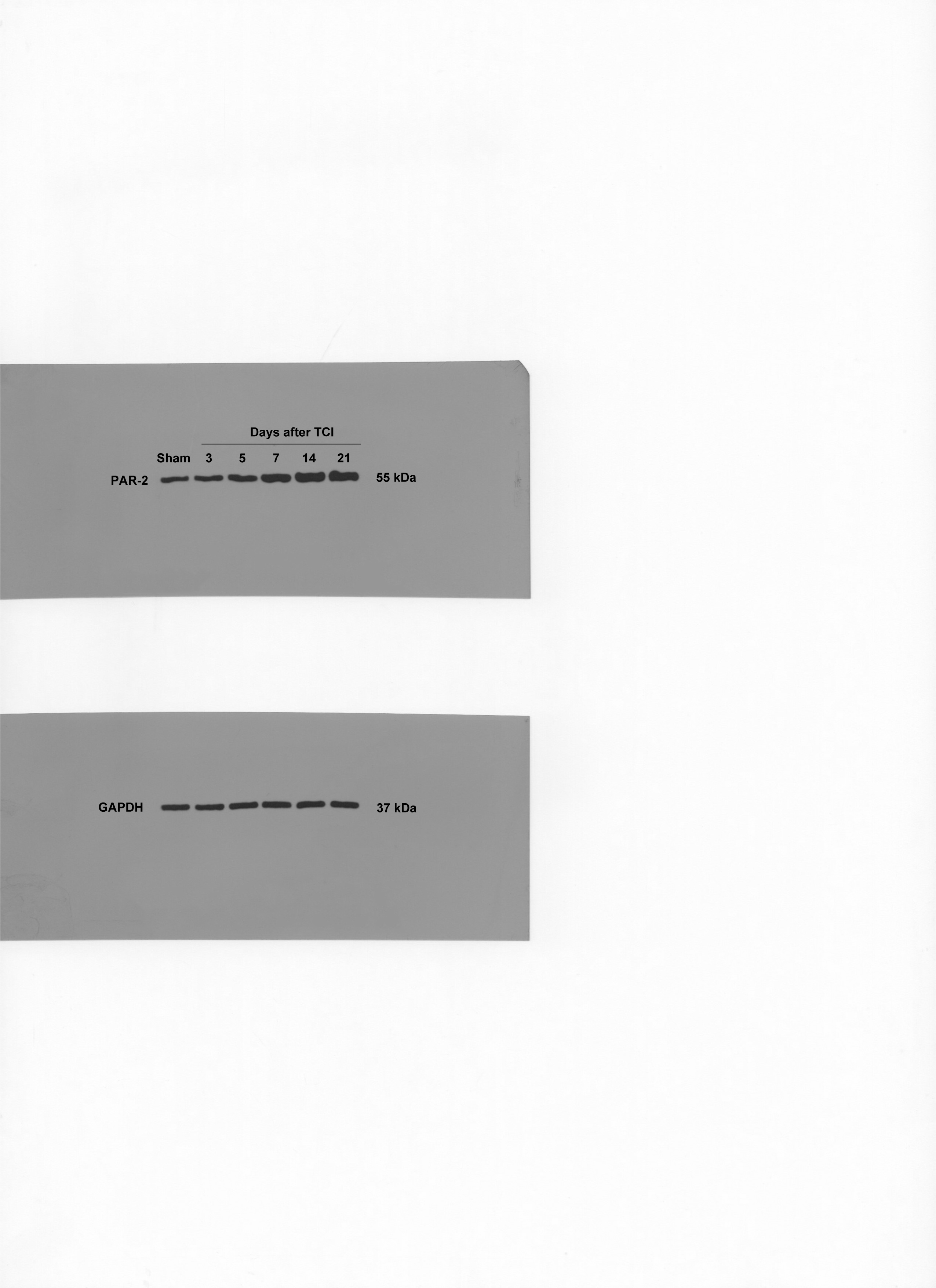

Supplement: Supplementary file 1 — Additional file 1. [file 12891_2022_5312_MOESM1_ESM.tif]

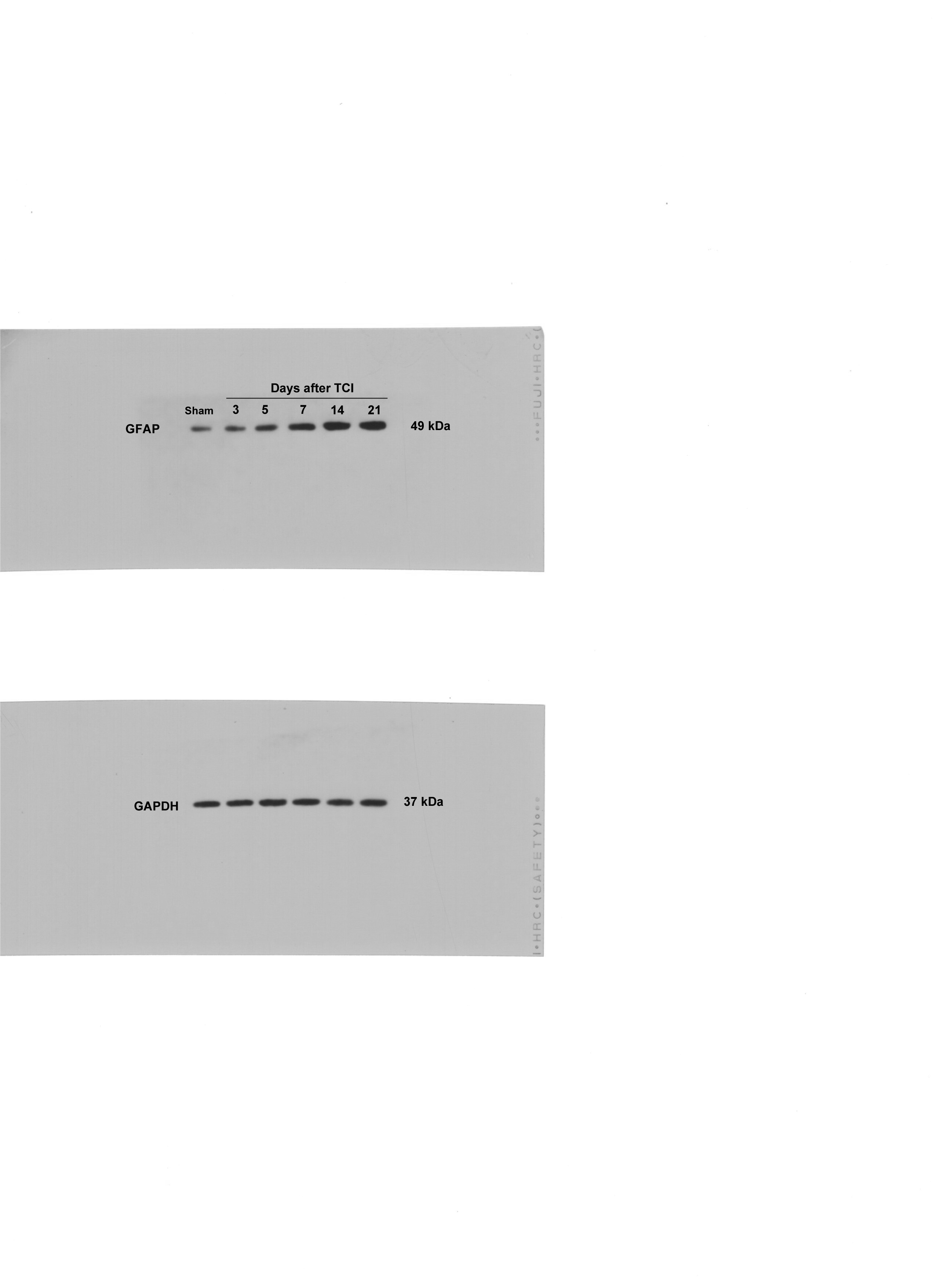

Supplement: Supplementary file 2 — Additional file 2. [file 12891_2022_5312_MOESM2_ESM.tif]

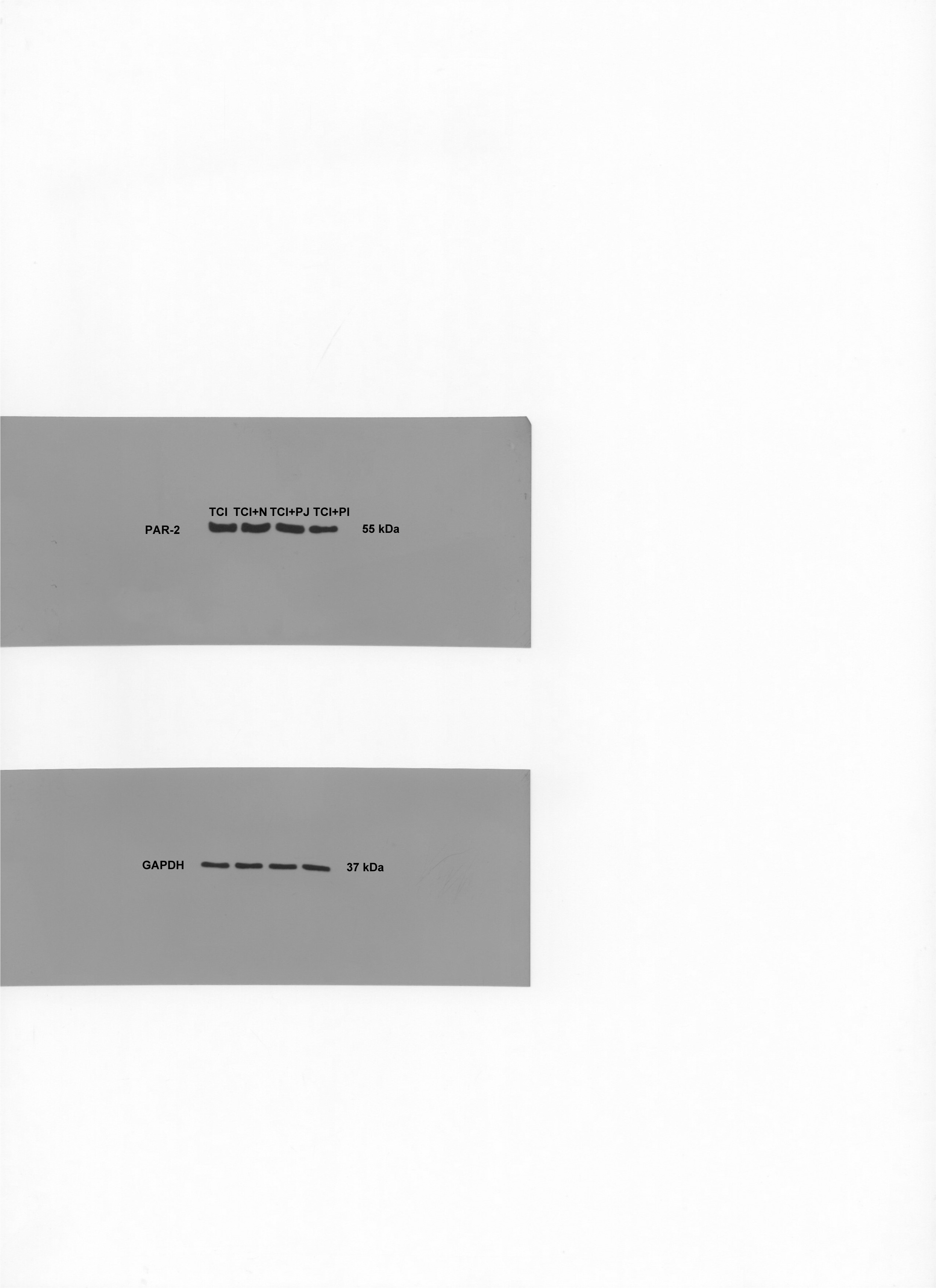

Supplement: Supplementary file 3 — Additional file 3. [file 12891_2022_5312_MOESM3_ESM.tif]

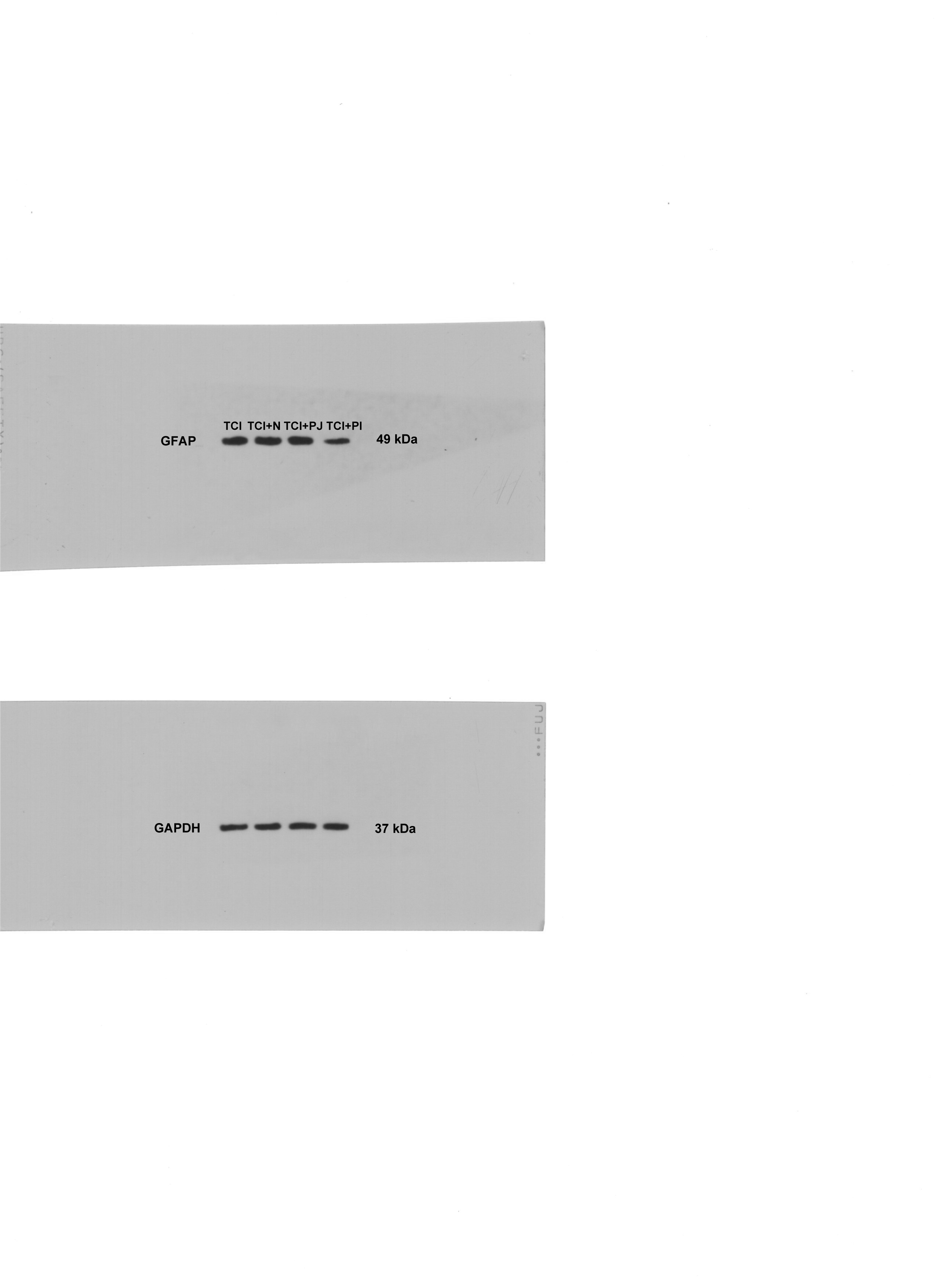

Supplement: Supplementary file 4 — Additional file 4. [file 12891_2022_5312_MOESM4_ESM.tif]

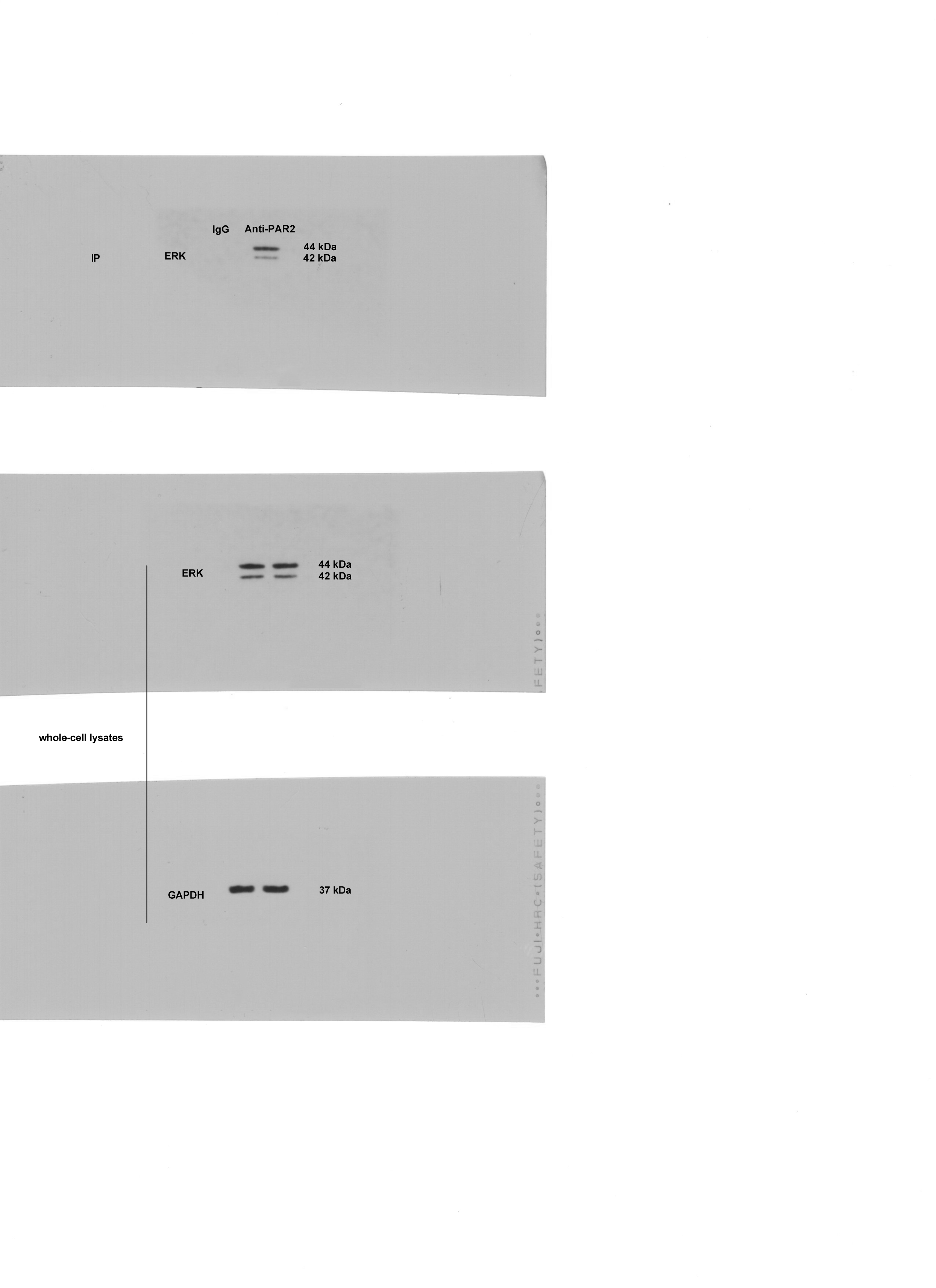

Supplement: Supplementary file 5 — Additional file 5. [file 12891_2022_5312_MOESM5_ESM.tif]

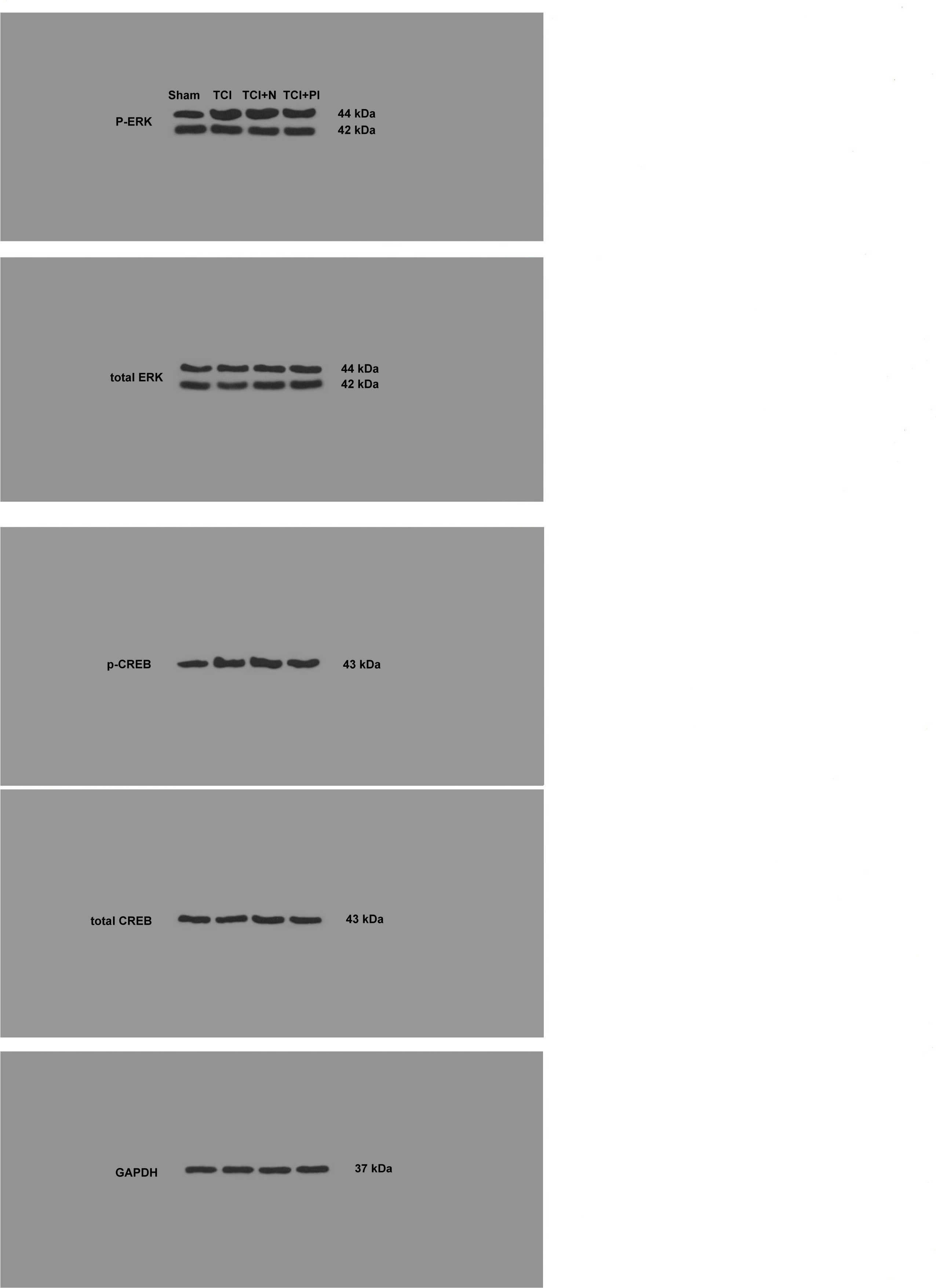

Supplement: Supplementary file 6 — Additional file 6. [file 12891_2022_5312_MOESM6_ESM.tif]

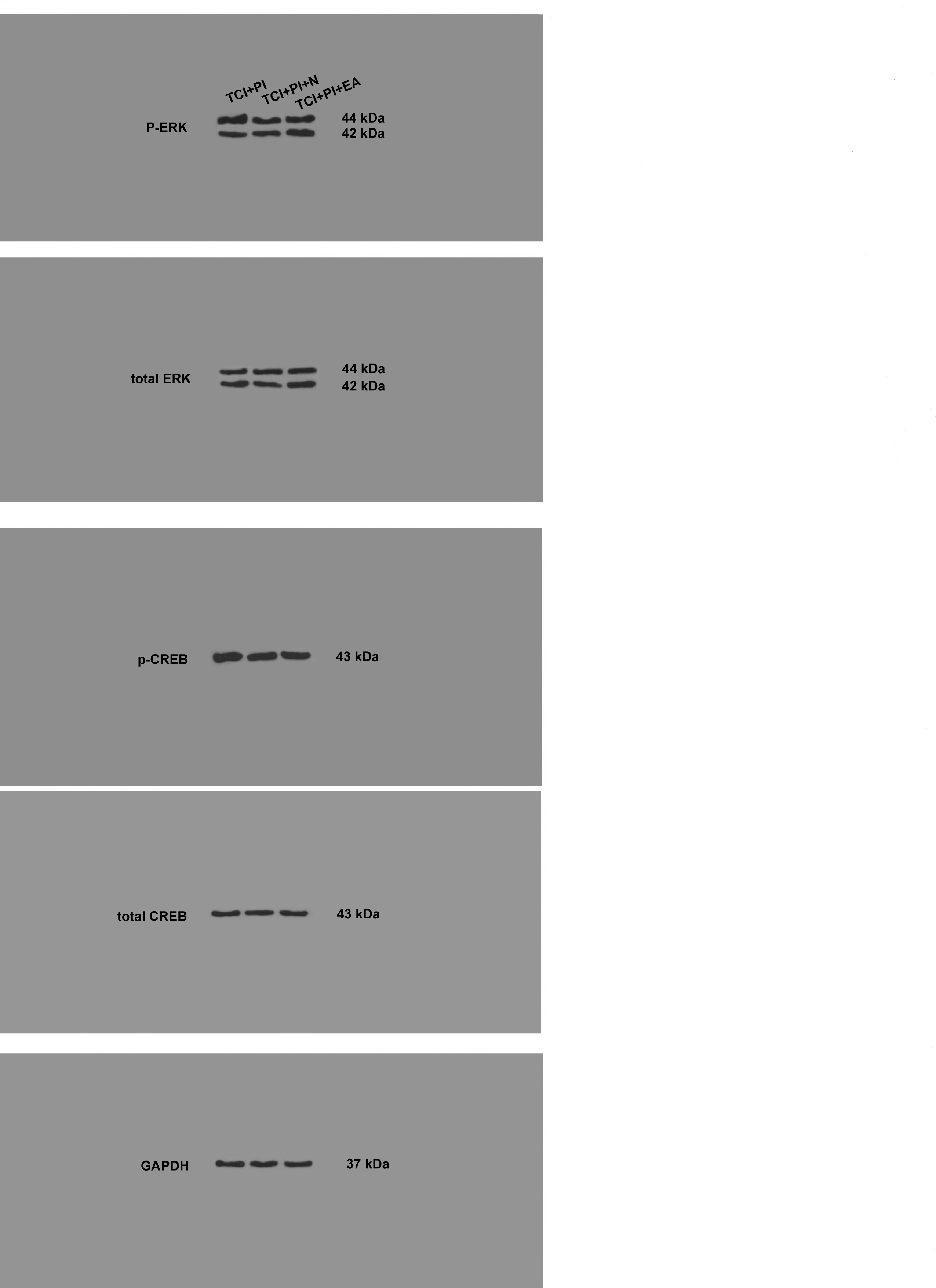

Supplement: Supplementary file 7 — Additional file 7. [file 12891_2022_5312_MOESM7_ESM.tif]

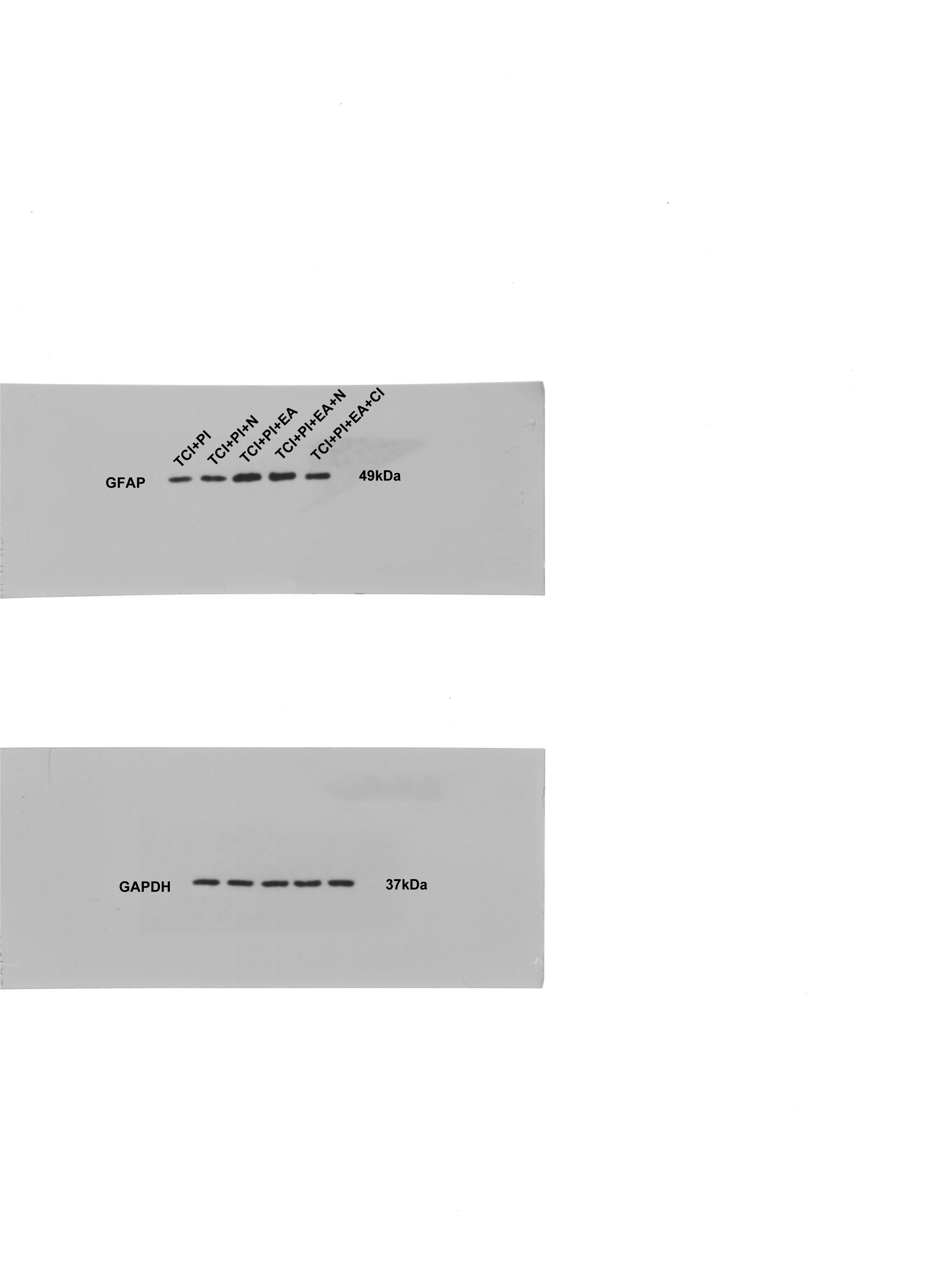

Supplement: Supplementary file 8 — Additional file 8. [file 12891_2022_5312_MOESM8_ESM.tif]
